# Structure and floristic composition associated with an endangered species *Beta patula* Aiton (Amaranthaceae) in the Islands of Madeira Archipelago

**DOI:** 10.3897/BDJ.9.e61091

**Published:** 2021-02-18

**Authors:** Humberto Nóbrega, Gregório Freitas, M. A. Zavattieri, Carla Ragonezi, Miguel Â. A. Pinheiro de Carvalho

**Affiliations:** 1 ISOPlexis, Centre of Sustainable Agriculture and Food Technology. University of Madeira. Campus da Penteada. 9020-105, Funchal, Portugal ISOPlexis, Centre of Sustainable Agriculture and Food Technology. University of Madeira. Campus da Penteada. 9020-105 Funchal Portugal; 2 Biology Department, Colégio da Mitra, University of Évora, Ap. 94. 7002-554, Évora, Portugal Biology Department, Colégio da Mitra, University of Évora, Ap. 94. 7002-554 Évora Portugal; 3 Institute of Earth Sciences (ICT), Colégio Luís António Verney, University of Évora. Rua Romão Ramalho, 59. 7002-554, Évora, Portugal Institute of Earth Sciences (ICT), Colégio Luís António Verney, University of Évora. Rua Romão Ramalho, 59. 7002-554 Évora Portugal; 4 CITAB Centre for the Research and Technology of Agro-Environmental and Biological Sciences, Vila Real, Portugal CITAB Centre for the Research and Technology of Agro-Environmental and Biological Sciences Vila Real Portugal; 5 Life Sciences Faculty, University of Madeira. Campus da Penteada. 9020-105, Funchal, Portugal Life Sciences Faculty, University of Madeira. Campus da Penteada. 9020-105 Funchal Portugal

**Keywords:** associated communities, crop wild relative, floristic survey, Macaronesian endemism

## Abstract

**Background:**

Twenty-two native Crop Wild Relatives (CWR) occur in specific dry environments of Madeira Archipelago, like Desembarcadouro islet in Ponta de São Lourenço and Chão islet in Desertas Islands. Nine of them share the same gene pool with crop species included in Annex I of the International Treaty on Plant Genetic Resources for Food and Agriculture. Amongst them, *Beta patula* Aiton, an IUCN Critically Endangered species, has been studied in detail for *in situ* and *ex situ* conservation. The present paper summarises the information recorded during the *Beta patula* population assessment. Valuable information on plant communities associated with this species was obtained.

**New information:**

The data provides information of a vegetation census spanning 7 years, from 2014 to 2020, in two uninhabited islets where *Beta patula* occurs, Desembarcadouro and Chão islets. The collected dataset consists of 1,786 vegetation descriptions, where 31 species were recorded. All generated data have been published and will be used towards the implementation of conservation actions and to establish a middle term management protocol for *Beta patula* and other CWR *in situ* conservation in the framework of a genetic reserve. This work is part of a EU LIFE Project, LIFE RECOVER NATURA and was conducted by members of the ISOPlexis Center, University of Madeira.

## Introduction

*Beta patula* has been classified as a Critically Endangered species by the International Union for Conservation of Nature (IUCN) in the Red List of Threatened Species (Pinheiro de Carvalho and Frese 2011), with an extent of occurrence of only 1.0 km² and its area of occupancy less than 0.80 km². In nature, the species is endemic to the Madeira Archipelago (Fig. 1a), more specifically occurring only in the Desembarcadouro and Chão islets. The Madeira Archipelago is part of the Macaronesia Region (Fig. 1b), a biogeographic area composed of four archipelagos in the North Atlantic Ocean (Azores, Canary Islands, Cape Verde and Madeira). From a geological point of view, all Macaronesian archipelagos are the consequences of mountain-forming and volcanic forces that occurred 60-70 million years ago (Fernández-Palacios et al. 2011). The contrasting landscapes and mild climate have created an ideal environment for a particularly rich biodiversity. The list of sites of community importance for the Macaronesian biogeographical region included in the Natura 2000 network, was the first to be adopted in December of 2001 and is updated every year. It contains 208 sites of community importance, covering over 5000 km² of land and sea. The list of sites of community importance for the Macaronesian biogeographical region can be found at: http://ec.europa.eu/environment/nature/natura2000/biogeog_regions/macaronesian/index_en.htm.

Amongst these sites are the Chão islet, included in the Desertas Islands (Natura 2000 site PTDES0001) and the Desembarcadouro islet, part of the Ponta de São Lourenço Peninsula (Natura 2000 site PTMAD0003). The habitat type classification of Desembarcadouro islet and Desertas Islands belongs to: a) vegetated sea cliffs with endemic flora from Macaronesian coast (EUNIS code 1250) and b) thermo-Mediterranean and pre-desert shrub (EUNIS code 5330) (EUNIS, European Nature Information System) (source: http://eunis.eea.europa.eu/sites/PTMAD0003; https://eunis.eea.europa.eu/sites/PTDES0001). Both ecosystems are of extreme importance, according to the Habitats Directive (Fig. 2). These areas harbour a wide variety of endemic and native plant species, some of them being crop wild relatives of relevant crops. More specifically, *Beta patula* is a crop wild relative of fodder, root and sugar beet crops. These species are included in Annex I of the International Threat for Genetic Resources for Food and Agriculture, for being important plant resources, ensuring world food security. Beet CWR have contributed to the breeding of sugar beet varieties, adding genes for resistance to biotic and abiotic stresses (Luterbacher et al. 2004; Panella et al. 2012; Capistrano-Gossmann et al. 2016). The source of these genes was obtained from accessions of CWR populations (Hajjar and Hodgkin 2007; Capistrano-Gossmann et al. 2016) showing their importance for *in situ* and *ex situ* conservation. In a recent work, Monteiro et al. 2018) pointed out the introgression of traits from CWR for decreasing biotic stresses constraints in sugar beets, namely using *Beta* and *Patellifolia* species, both having disease-resistant characteristics.

Although *Beta patula* occurs in protected islets, it is threatened by introduced animals, particularly by rabbits and seagull populations, possibly causing a severe fragmentation of the population and a continuous decline in the extent and quality of its habitat. Therefore, there was a need for the development and implementation of a species action plan and a special site management plan (Frese et al. 2010, Pinheiro de Carvalho et al. 2010, Pinheiro de Carvalho et al. 2012, Maxted et al. 2012 and Frese et al. 2012). Consequently, *B. patula* was one of the target species of AEGRO project (An Integrated European In Situ Management Work Plan: Implementing Genetic Reserves and On Farm Concepts - AGRI GENRES 057), whereupon the ISOPlexis team started, in 2008, the study to establish *B. patula* distribution, population baseline and to set up appropriate strategies for this CWR conservation. The project also included the monitoring of ecogeographic conditions (habitat description; altitudinal gradient; soil type and edaphic conditions, climatic parameters) that can limit the species distribution or be a factor of plant diversity differentiation. The plant population survey aims to set up its boundaries and estimate the patches of plant coverage and population sizes. Since then, a continuing effort has been made to validate *B. patula* conservation *in situ* and *ex situ* by the storage of accessions (ISOP 2512 and ISOP 1911) in the ISOPlexis Gene Bank, at the University of Madeira and to validate population sizes and dynamics. These studies include a complete floristic analysis at the selected occurrence locations of *B. patula* in Desembarcadouro islet (DI) and Chão islet (CI). This work was financed by LIFE RECOVER NATURA - LIFE12 NAT / PT / 000195 coordinated by the Madeira Forestry and Nature Conservation Institute IP-RAM (IFCN IP-RAM). The results of a monitoring system to control the conservation status of *Beta patula* were published in Nóbrega et al. 2020.

This work condenses the results of a 7 year floristic study of all species associated with *B. patula*. The floristics analysis is still ongoing and it will continue, thus contributing to the permanent monitoring that aims to determine any signs of change in the diversity of these sites, due to climate change, animal pressure or any other interference in habitat that may affect diversity and long-term conservation of the endangered *B. patula* and other important endemisms in its distribution areas.

## General description

### Purpose

The LIFE RECOVER NATURA - *Beta patula* population assessment consisted in the use of previous information obtained during AEGRO project to monitor the populations dynamics and validate the established species baseline with the ultimate goal of implementing a genetic reserve and its management plan. With this work, it was also possible to make a vegetation classification, species trend analysis, distribution maps of plant communities and Natura 2000 habitat types for the occurrence areas of *Beta patula* (Suppl. material 1).

## Project description

### Title

LIFE RECOVER NATURA - LIFE12 NAT / PT / 000195

### Personnel

Humberto Nóbrega, Gregório Freitas and Miguel Ângelo Almeida Pinheiro de Carvalho.

### Study area description

The study area comprises two Nature 2000 sites located at the Madeira Archipelago, Portugal. The Chão islet, included in the Desertas Islands (PTDES0001) and the Desembarcadouro islet, a part of the Ponta de São Lourenço Peninsula (PTMAD0003). Both ecosystems are of extreme importance, with reference to the Habitats Directive 1250 - Scarps with endemic flora from Macaronesian Coastlines and 5330 - Pre Desertic thermo-mediterranean Shrubland.

### Design description

The objective of Action A6 was to determine occurrence areas and effective population size for the endangered and endemic *Beta patula* Aiton species. This action was part of a LIFE Project entitled LIFE RECOVER NATURA – Recovery of the species and land habitats of the Natura 2000 sites of Ponta de São Lourenço and Desertas Islands.

### Funding

This Project was funded by the EU LIFE Programme

## Sampling methods

### Sampling description

**Figure 3.**
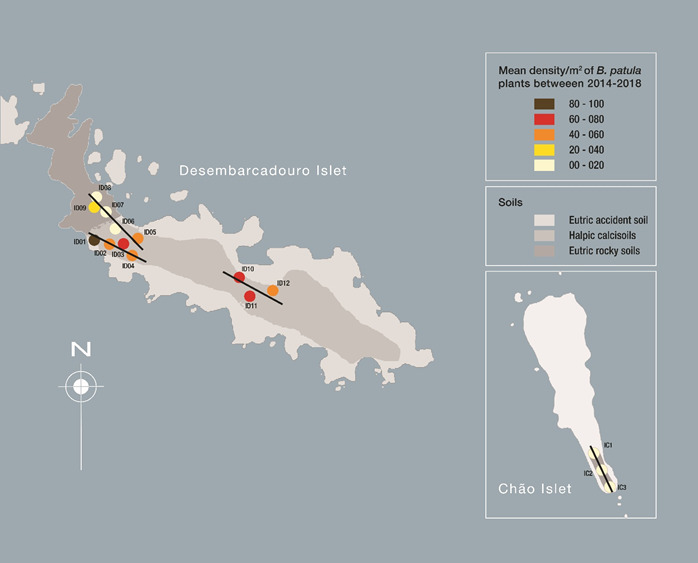
Location of the 15 quadrants established for species survey, sampling and monitoring. Sampling sites DI1 to DI12 correspond to the Desembarcadouro islet and CI1 to CI3 are indicated for the Chão islets. Soil types in the Desembarcadouro islet are described. No information exists on the soil types for Chão islet. On both islets, the colours attributed to the circles for each sampling site correspond to a mean density of *B. patula* plants (plants X square metre).

To collect the floristic data, 15 areas were selected, taking into consideration that these areas are within the *Beta patula* distribution range and have a good resolution for herbaceous and small bush plants, being that the floristic composition falls in such criteria (Fig. 3), 12 in the Desembarcadouro islet (DI01; DI02; DI03…DI12) and three in the Chão islet (CI01; CI02 and CI03). These areas are quadrats with 16 m^2^ (4 x 4 m) facing North (so replicability could be ensured for each successive sampling) displayed along three parallel transects defined in Desembarcadouro. Inside each quadrat, a full counting and identification of all individuals was carried out in four randomly-selected square metres, using the Research Randomizer Generated Numbers software (Urbaniak and Plous 2013). Sampling effort was dependent on plant density. Plant identification was done according to Pires and Fontinha (2008), Aguiar et al. (2004) and Press and Short (1994). The assigned names of all taxa cited in this work follow the nomenclature given by the Plant List (http://www.theplantlist.org/). Soil types in the Desembarcadouro islet are according to Pinto et al. (1992). No information on the soil types in Chão islet exists in literature.

**Figure 4.**
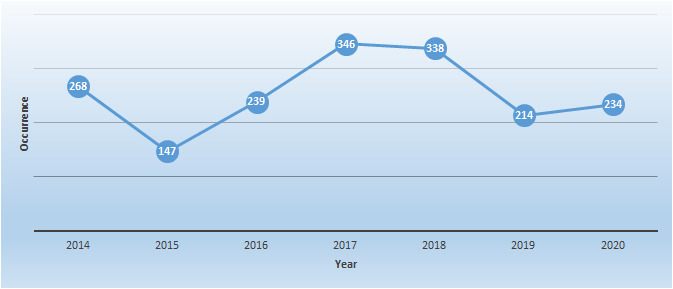
Summary of number of species occurrences for all 15 sampling sites from 2014 to 2020.

The 16 m^2^ plots were set up at a minimum distance from each other of approximately 50 m. Final specimen counts represent the average results of two separate counts per site. If both counts were significantly different, a re-count was made. Each sampling plot was also marked with a coloured steel rod and geographic coordinates were taken with the help of a Global Positioning System (GPS) to facilitate the identification and retrieval of the plots for future monitoring. The target species *B. patula* and other beet CWR were particularly marked, as well as the Madeiran and Macaronesian endemisms. The species occurrence, floristic composition and structure were studied. The floristic composition of DI and CI sites are similar, due to geographic proximity and similar climate and soil conditions. Plant species were grouped in 13 families. Thirty-one distinct plant species were recorded during the study; 25 species were found in DI and 12 in CI. Five of these species, excluding the target species, *B. patula*, were common to both sites and recorded in occupancy areas, namely *Mesembryanthemum crystallinum* L., *Mesembryanthemum nodiflorum* L., *Suaeda vera* Forssk. ex J.F. Gmel, *Spergula fallax* (Lowe) E.H.L. Krause and *Crepis divaricata* (Lowe) F.W. Schultz. The species were also classified as introduced or probably introduced (three taxa) and native or probably native (19 taxa). Amongst the endemic species, nine taxa were present. Of those, five taxa are endemic to Madeira Islands and four taxa are endemic to the Macaronesia Region (Suppl. material 1). Fig. 4 shows the summary number of species occurrence in the 15 sampling sites and its annual variation, in the time period between 2014 and 2020. The graph shows that 2017 was the year with the highest number of species accounted, followed very closely by the year 2018.

The tree map analyses the floristic composition and structure of the habitats where *Beta patula* occurs. Fig. 5 shows the relative species frequence occurring in Desembarcadouro and Chão islets. The frequency and dominance were established, based on total account of specimens. The *Mesembryanthemum crystallinum* is the most predominant species in both islets, while *Beta patula* is the third most common.

Fig. 6 shows the relative frequency of families occurring in Desembarcadouro and Chão islets. The most frequent families are Amaranthaceae represented by six species and Aizoaceae represented by four species. The Amaranthaceae family dominates in Desembarcadouro islet and the Aizoaceae dominates in the Chão islet.

## Geographic coverage

### Description

The general coverage area comprises two islets that are part of the Madeira Archipelago, Portugal. The Desembarcadouro islet is located on the eastern peninsula of Madeira Island (Ponta de São Lourenço) and the Chão islet is one of the three islets that compose the Desertas Islands.

### Coordinates

32°34'30''N and 32°44'27.6''N Latitude; 16°40'44.4''W and 16°32'20.4''W Longitude.

## Taxonomic coverage

### Description

The floristic composition of Desembarcadouro islet (DI) and Chão islet (CI) both Macaronesian sites, have similarities, due to their geographic proximity and similarity of soil composition and climate conditions. The flora composition of Desembarcadouro islet is part of the *Mayteno umbellatae*-*Oleion maderensis* Vegetation Series Complex (Aguiar et al. 2004, Costa et al. 2012.) It is characterised as a dry infra-mediterranean series, exclusive of southern rocky cliffs, restricted to 0-200 m above sea level. On the sampling areas, two vegetation mosaic communities stand out: the arid to semi-arid infra-mediterranean halinotrophyle community of Ponta de São Lourenço and *Calendula maderensis*-*Suaedetum verae*, composed of species, such as *Suaeda vera* Forssk. ex J. F. Gmel., *Calendula maderensis* DC (synomym of Calendula incana subsp. maderensis (DC.) Ohle), *Lotus glaucus* Sol., *Chenoleoides tomentosa* (Lowe) Bostch. (synonym of *Bassia tomentosa* (Lowe) Maire & Weiller), amongst others. The other vegetation mosaic is the semi-arid infra-mediterranean halinotrophyle community of Madeira and Porto Santo *Senecio incrassate*-*Mesembryanthemum cristalini*, composed of species, such as *Mesembryanthemum crystallinum* L., *Mesembryanthemum nodiflorum* L., *Senecio incrassatus* Lowe, *Aizoon canariensis* L., *Tetragonia tetragonioides* (Pall.) Kuntze and *Spergula fallax* (Lowe) E.H.L. Krause (Fig. 7).

### Taxa included

**Table:** 

Rank	Scientific Name	Common Name
kingdom	Plantae	Plants
phylum	Tracheophyta	
class	Magnoliopsida	Dicotyledons
class	Liliopsida	Monocotyledons
order	Asparagales	
order	Asterales	
order	Brassicales	
order	Caryophyllales	
order	Fabales	
order	Lamiales	
order	Malpighiales	
order	Malvales	
order	Poales	
family	Aizoaceae	
family	Amaranthaceae	
family	Asteraceae	
family	Brassicaceae	
family	Caryophyllaceae	
family	Euphorbiaceae	
family	Fabaceae	
family	Frankeniaceae	
family	Malvaceae	
family	Plantaginaceae	
family	Poaceae	
family	Polygonaceae	
family	Xanthorrhoeaceae	

## Temporal coverage

**Data range:** 2014-4-29 – 2020-8-10.

## Usage licence

### Usage licence

Creative Commons Public Domain Waiver (CC-Zero)

## Data resources

### Data package title

*Beta patula* Population Assessment from 2014 to 2020 in Madeira Archipelago, Portugal

### Resource link

https://www.gbif.org/dataset/004ef655-c4ec-4b45-8c00-23603799af15

### Alternative identifiers

https://doi.org/10.15468/j3qfk5

### Number of data sets

1

### Data set 1.

#### Data set name

*Beta patula* Population Assessment from 2014 to 2020 in Madeira Archipelago, Portugal

#### Data format

Darwin Core Archive format

#### Number of columns

35

#### Character set

UTF-8

#### Download URL

http://ipt.gbif.pt/ipt/archive.do?r=isoplexis_beta_patula

#### Data format version

1.8

#### 

**Data set 1.** 

Column label	Column description
id	Unique ID for each occurrence record.
type	Type of the record, as defined by the Public Core standard.
language	The language used in the observation details.
licence	Reference to the licence under which the record is published.
rightsHolder	The rights holder to whom the data belong.
datasetID	A unique identifier to this dataset.
institutionID	The identity of the institution publishing the data.
ownerInstitutionCode	The code of the institution who owns publishing data rights
institutionCode	The code of the institution publishing the data.
datasetName	The name of this dataset.
parentEventID	An identifier for the broader Event that groups this and potentially other Events.
eventID	Identifier of the events, unique for the dataset.
samplingProtocol	The sampling protocol used to observe the species.
eventDate	The date-time or interval during which an Event occurred. For occurrences, this is the date-time when the event was recorded.
sampleSizeUnit	The unit of measurement of the size of a sample in a sampling event.
sampleSizeValue	A numeric value for a measurement of the size of a sample in a sampling event.
habitat	A textual description of the habitat in which the taxon was observed.
higherGeographyID	The code of the region in which the observation occurred. Here: http://vocab.getty.edu/tgn/7003831
higherGeography	A description of the region in which the observation occurred.
georeferencedBy	A list (concatenated and separated) of names of people who determined the georeference for the Location.
continent	The name of the continent in which the location occurs.
country	The name of the country in which the location occurs.
countryCode	The ISO 3166-1-alpha-2 country code.
islandGroup	The name of the island group in which the location occurs.
island	The name of the island in which the location occurs.
stateProvince	The first-level administrative subdivision of the country in which the observation occurred.
municipality	The name of the municipality in which the location occurs.
locality	The name of the locality in which the location occurs.
decimalLatitude	The geographic latitude in decimal degrees of the geographic centre of a location.
decimalLongitude	The geographic longitude in decimal degrees of the geographic centre of a location.
geodeticDatum	The ellipsoid, geodetic datum or spatial reference system (SRS) upon which the geographic coordinates given in decimalLatitude and decimalLongitude are based. Here: WGS84
minimumElevationInMetres	The lower limit of the range of elevation (altitude, usually above sea level), in metres.
coordinateUncertaintyInMetres	Indicator for the accuracy of the coordinate location, described as the radius of a circle around the stated point location in metres. Here: 3 metres
footprintWKT	A Well-Known Text (WKT) representation of the shape that defines the location.
footprintSRS	The spatial reference system (SRS) upon which the geographic coordinates given in decimalLatitude and decimalLongitude are based. Here: EPSG:4326

## Supplementary Material

93A2777A-6EDC-5109-A09C-BFB349A5DB2D 10.3897/BDJ.9.e61091.suppl1 Supplementary material 1 List of the species (Spermatophytes) and number of individuals surveyed between 2014 and 2020 in Desembarcadouro (DI) and Chão (CI) islets Data type Occurrences Brief description List of the species (Spermatophytes) surveyed between 2014 and 2020 in Desembarcadouro (DI) and Chão (CI) islets. The species are classified by their family affiliation and their status. Taxonomic names are in accordance with The Plant List 1.1 (2013) http://www.theplantlist.org/. The number of individuals counted per specie/year is also presented as well as the total number per islet in the 7 years of sampling. File: oo_491178.txt https://binary.pensoft.net/file/491178 Humberto Nóbrega, Gregório Freitas, M. A. Zavattieri, Carla Ragonezi, Miguel Â. A. Pinheiro de Carvalho

## Figures and Tables

**Figure 1a.**
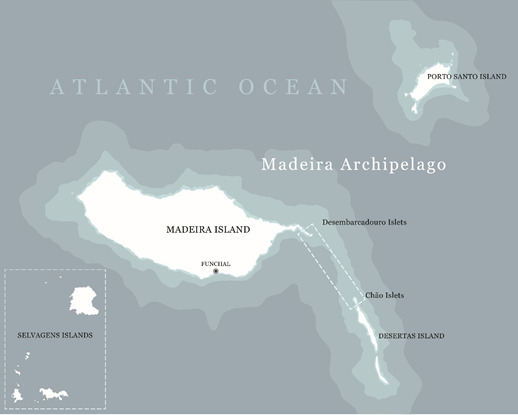
Madeira Archipelago

**Figure 1b.**
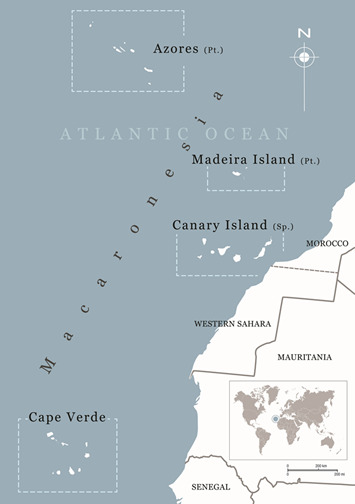
Macaronesian archipelagos

**Figure 2a.**
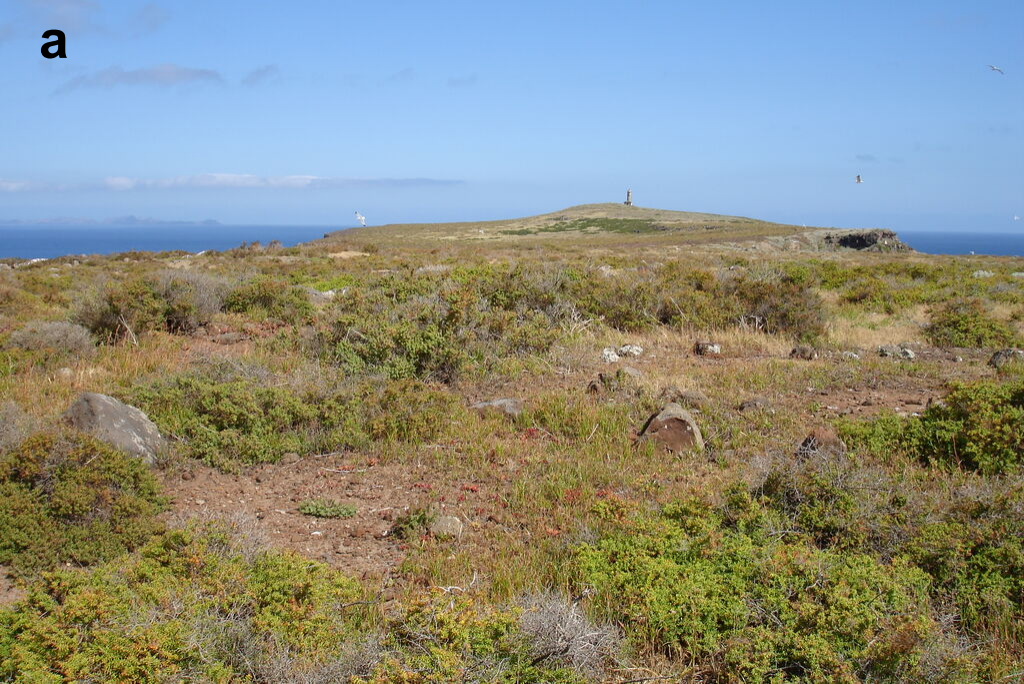
Chão islet habitat aspect. In the distance, it is possible to see Desembarcadouro islet.

**Figure 2b.**
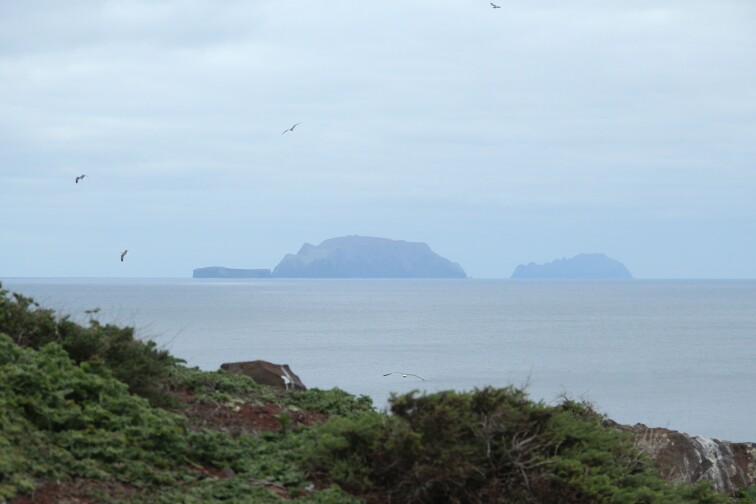
View of Chão islet (flat islet on the left) from the Desembarcadouro islet.

**Figure 2c.**
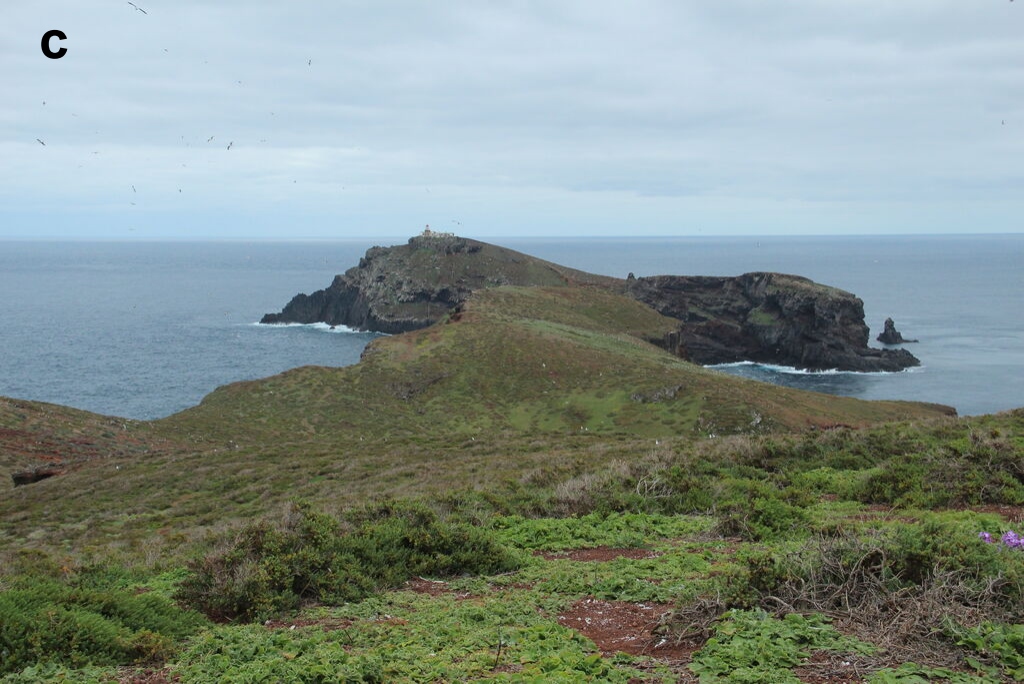
General vegetation aspect of Desembarcadouro islet's habitat, with Farol islet in the background.

**Figure 2d.**
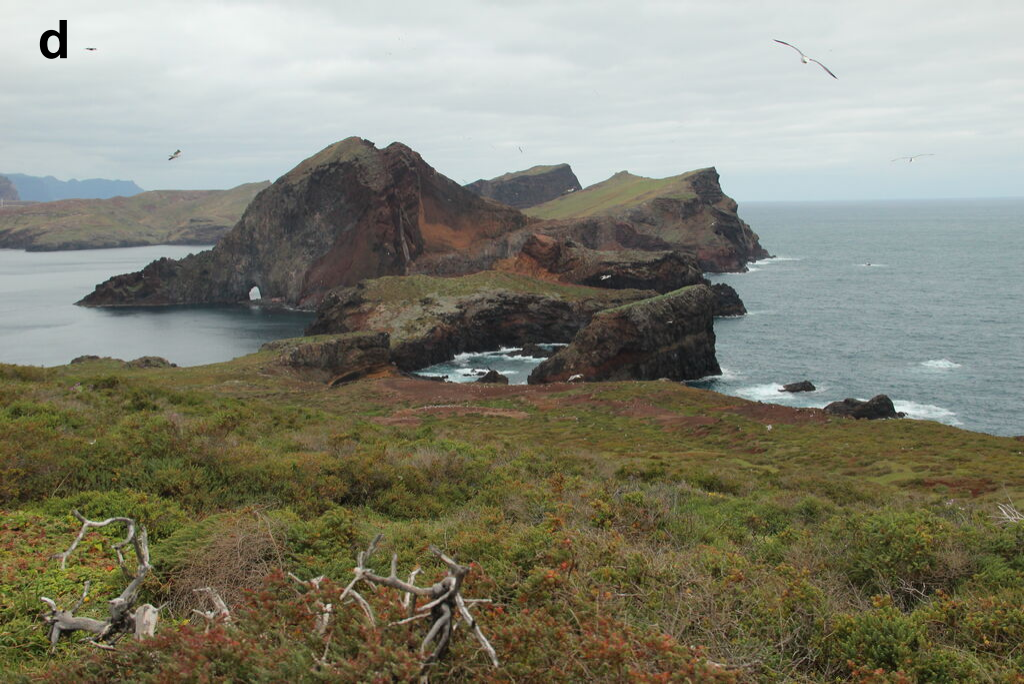
General vegetation aspect of Desembarcadouro islet's habitat, with Ponta de São Lourençao in the background.

**Figure 5a.**
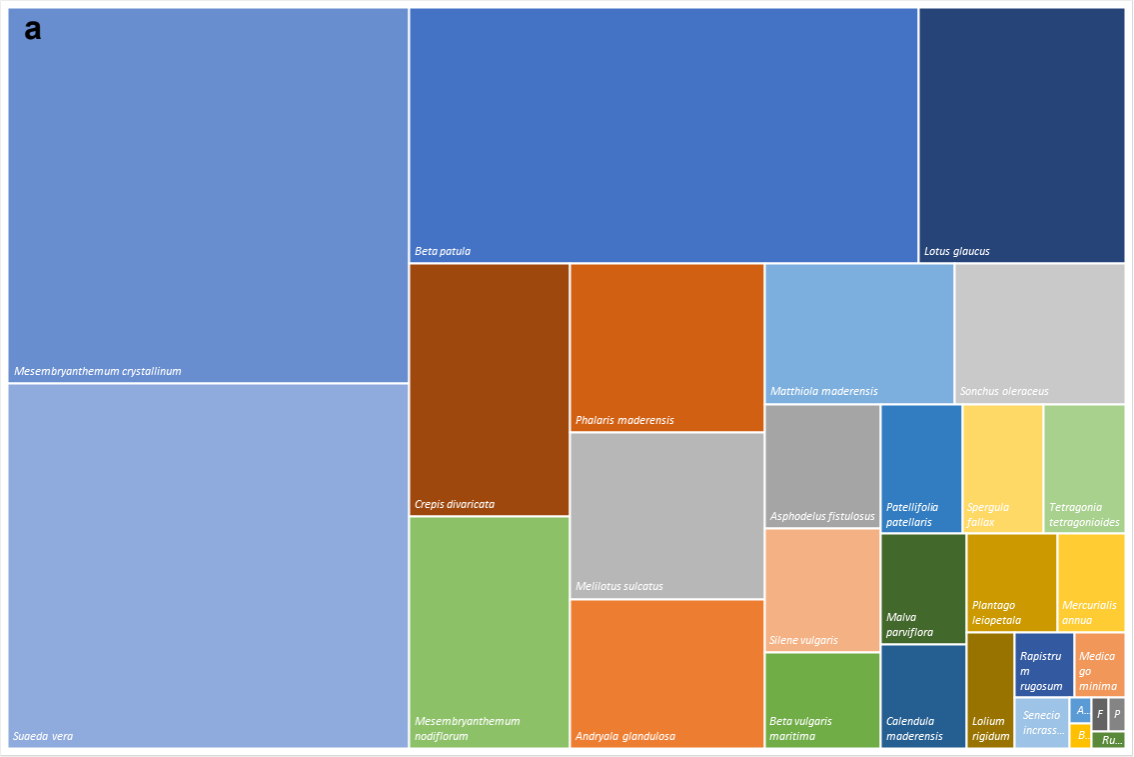
Desembarcadouro islet species structure.

**Figure 5b.**
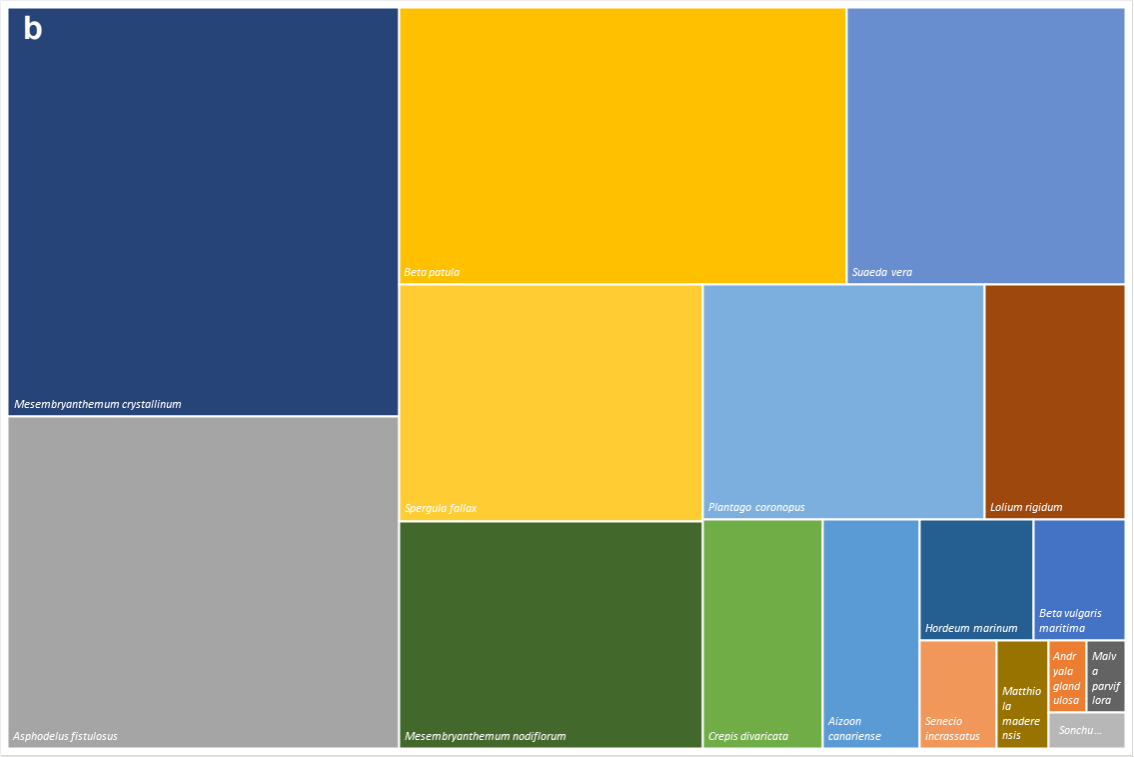
Chão islet species structure.

**Figure 6a.**
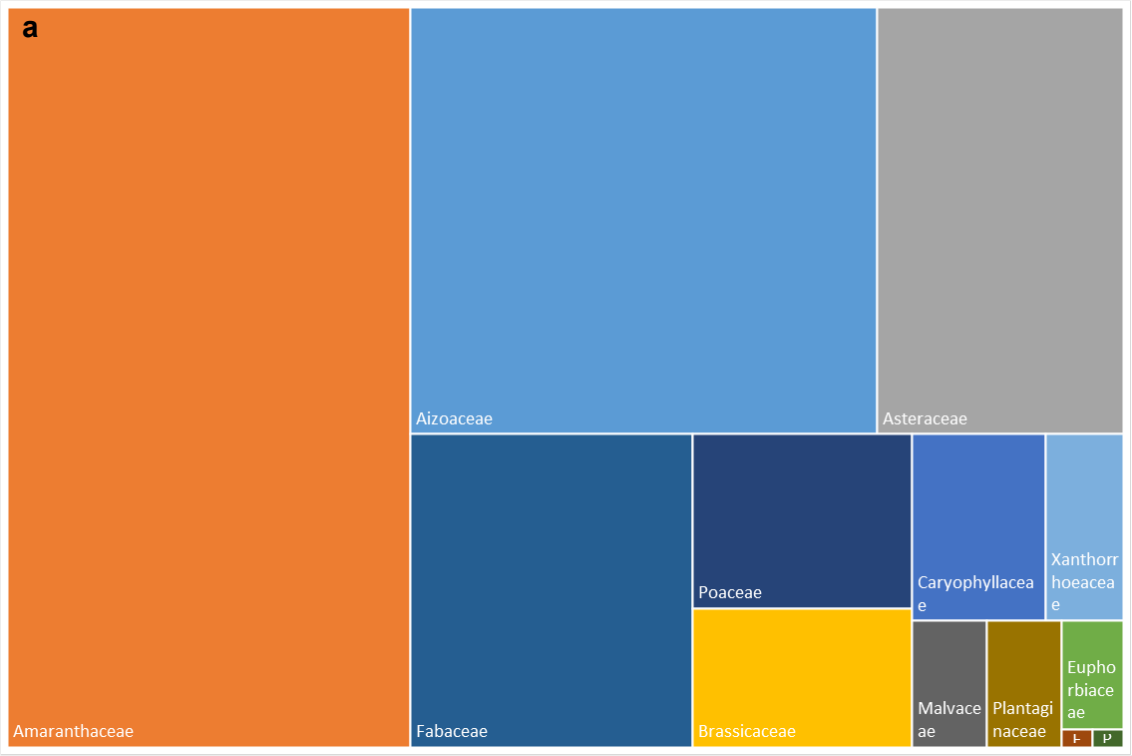
Most represented families of Desembarcadouro islet from 2014 to 2020.

**Figure 6b.**
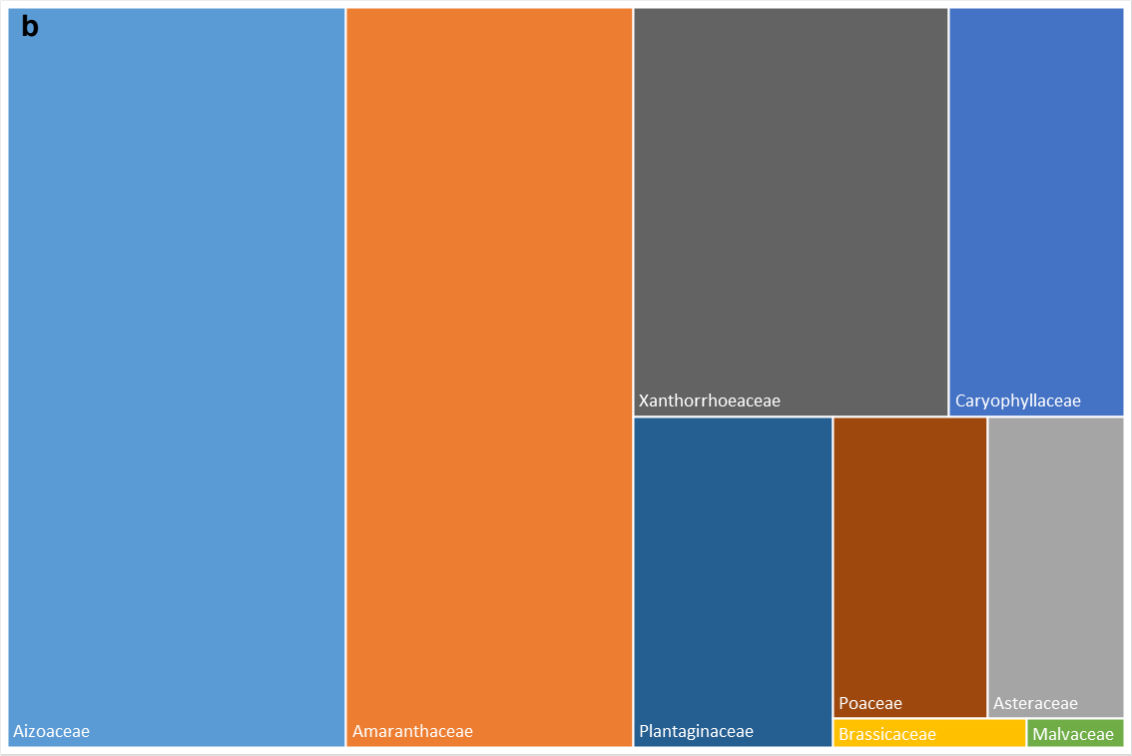
Most represented families of Chão islet from 2014 to 2020.

**Figure 7a.**
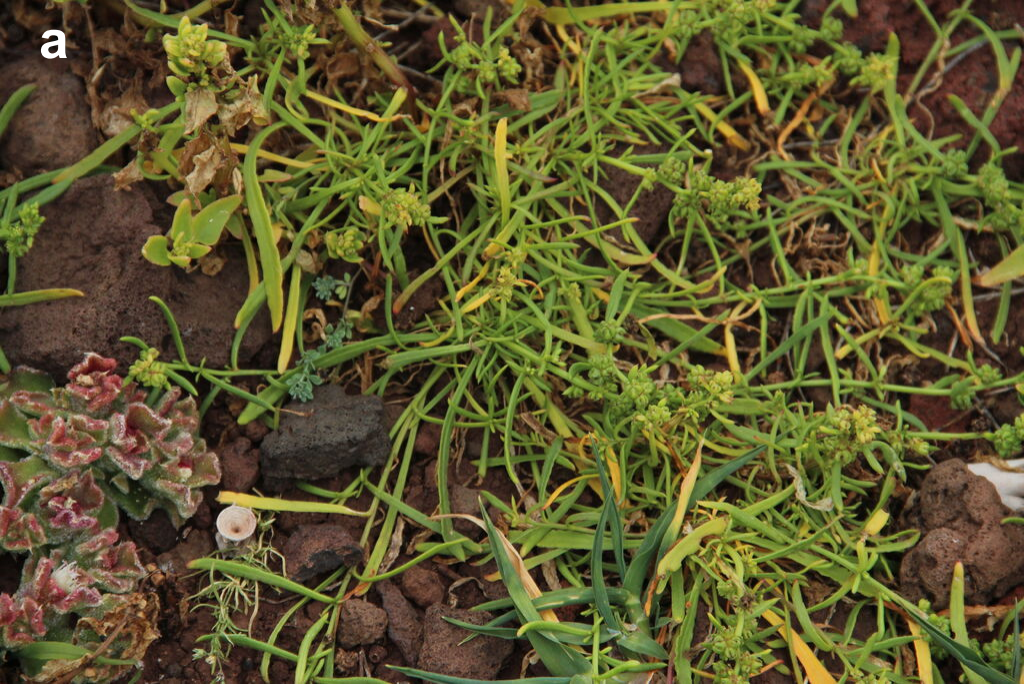
*Beta patula* Aiton

**Figure 7b.**
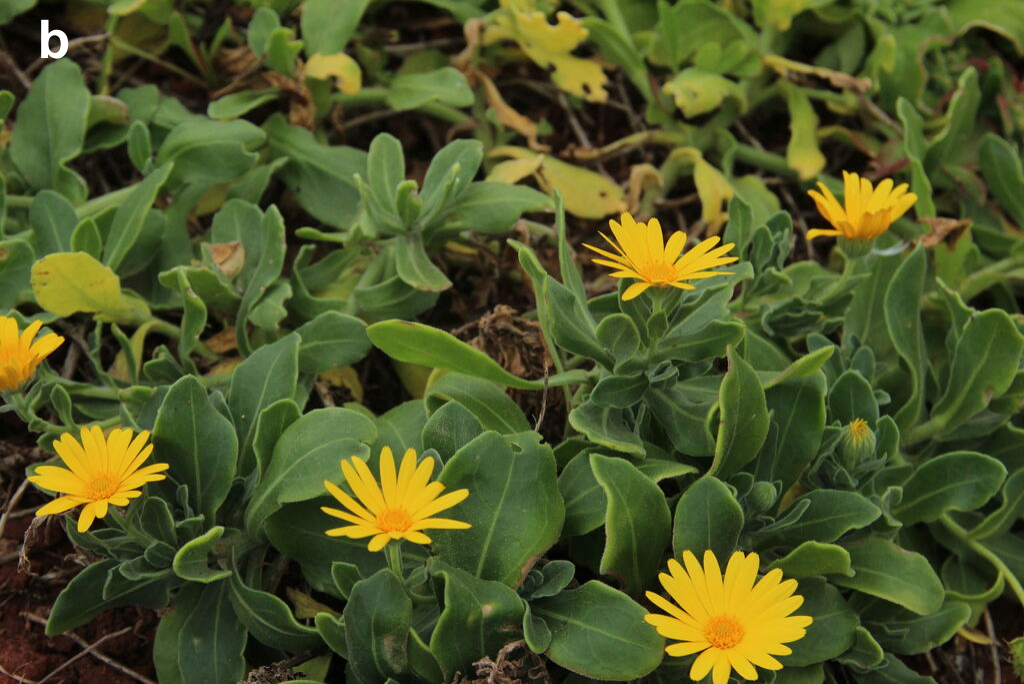
Calendula incana subsp. maderensis (DC.) Ohle

**Figure 7c.**
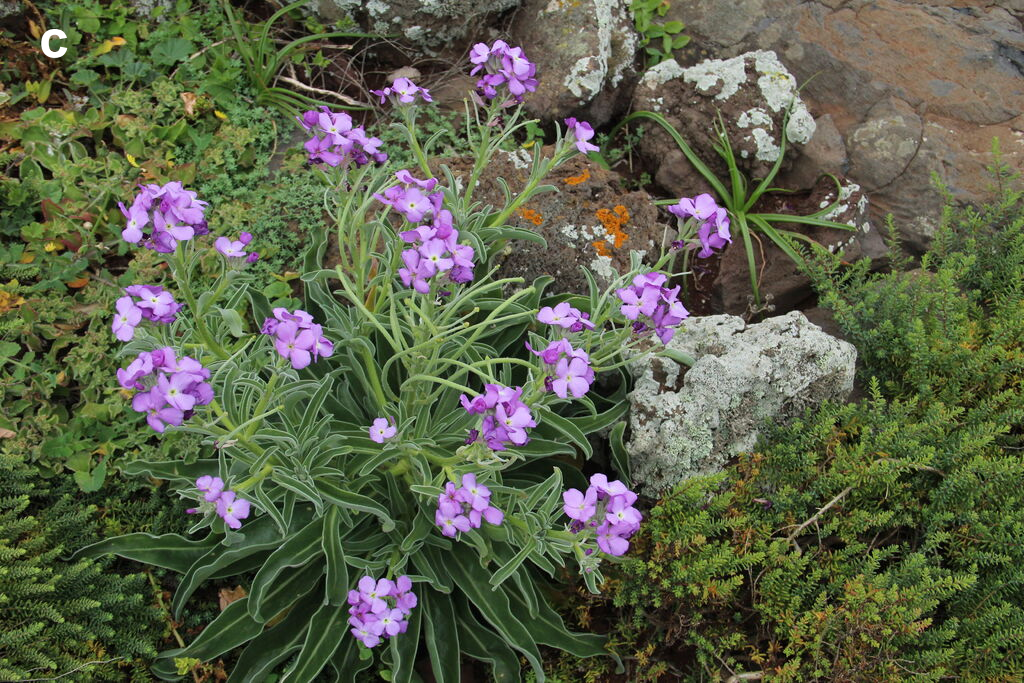
*Matthiola maderensis* Lowe

**Figure 7d.**
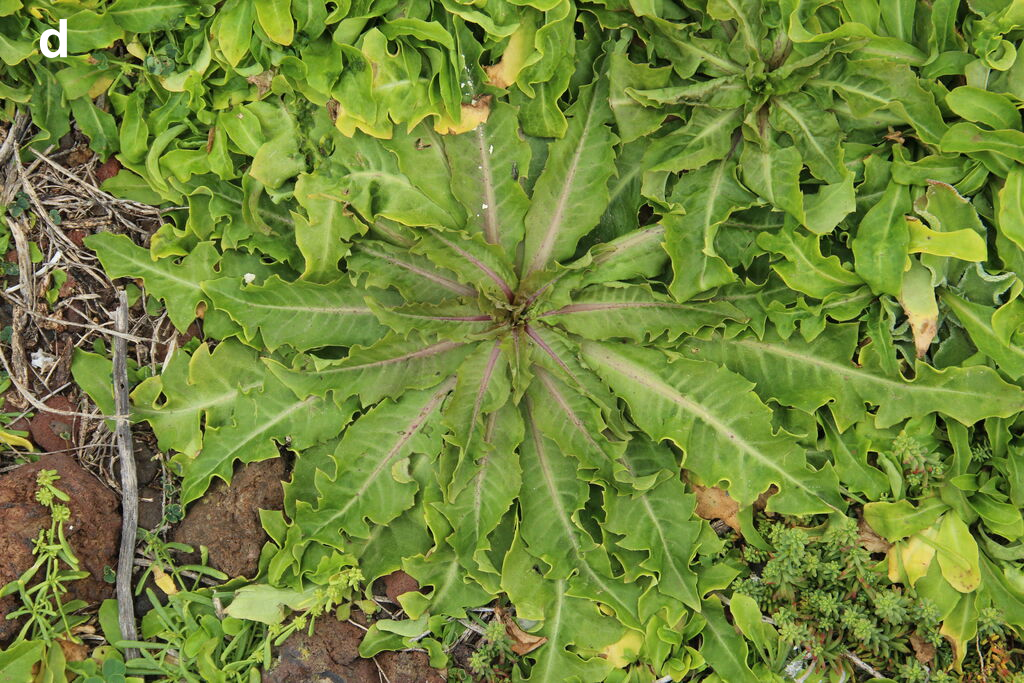
*Crepis divaricata* (Lowe) F.W. Schultz
